# Optimizing Digital Solutions to Improve Access to Comprehensive Primary Health Care Services in Remote Indigenous Communities: Protocol for a Participatory Action Research Project

**DOI:** 10.2196/68892

**Published:** 2025-09-17

**Authors:** Vishnu Khanal, Emily Saurman, Deborah J Russell, Nicki Newton, Karina Coombes, Alexandar Puruntatameri, Sarah Norris, Amy von Huben, Tamsin Cockyane, Paul Burgess, John Wakerman, Timothy Shaw

**Affiliations:** 1 Remote Health Systems and Climate Change Centre Menzies School of Health Research Charles Darwin University Alice Springs Australia; 2 Sydney School of Public Health University of Sydney Sydney Australia; 3 Faculty of Medicine and Health University of Sydney Sydney Australia; 4 Leeder Centre for Health Policy, Economics and Data School of Public Health, Faculty of Medicine and Health University of Sydney Sydney Australia; 5 Department of Health Northern Territory Government Darwin Australia

**Keywords:** Indigenous peoples, primary health care, digital health, telehealth, remote, access, participatory action research, co-design, Indigenous health

## Abstract

**Background:**

Aboriginal and Torres Strait Islander (Indigenous) peoples living in remote Australia experience a heavy burden of ill health and multiple barriers to accessing health care. Digital health technologies (DHTs) have the potential to help overcome some of these challenges and increase access to comprehensive primary health care (CPHC), thereby improving equity of health outcomes. However, little is known about the community and provider preferences for the use of digital technologies for improving health and wellness.

**Objective:**

The study aims to co-design, implement, and evaluate how DHTs can improve access to CPHC in remote Indigenous communities in the Northern Territory (NT), Australia.

**Methods:**

This multiphased project will take a participatory action research approach to co-design and optimize digital health solutions with local community members and health service staff in 2 communities. Our mixed methods approach will include pre- and postimplementation focus group discussions, interviews, quantitative analysis of CPHC utilization administrative data, and surveys administered by Indigenous community-based researchers to understand the use of digital devices and connectivity, eHealth literacy, preferences for different attributes of DHTs using best-worst scaling, and consumer satisfaction and experiences with DHT interventions. Priority DHTs will be selected for implementation based on consumer and health staff preferences. Focus group discussions and interview data will explore community and health service staff preferences, experiences, and satisfaction with implemented DHTs. A realist approach will be taken to identify how DHT interventions work, for whom, and in what circumstances, so that the understanding of why some interventions work while others do not is expanded. Economic analyses will be conducted to calculate the incremental costs and benefits of implemented DHT interventions. The scalability of digital health solutions will be tested in two additional communities. Project partners include key funding, service, and support agencies in the NT and nationally.

**Results:**

As of November 2024, we have selected two implementation sites. Digital health initiatives are underway at the implementation sites, and evaluation activities are progressing. The initial findings from these sites have informed our scalability assessment in an additional two sites.

**Conclusions:**

Knowledge translation is integral to the study design, which involves partnering with consumers, CPHC service providers, and a range of key stakeholders to inform health service providers and policy makers about which DHTs work for which groups of consumers, and under what circumstances, to improve access to CPHC. This unique study will accommodate consumer and provider preferences regarding the use of DHTs to improve CPHC access and address the lack of knowledge about how to deploy digital solutions to best support CPHC in remote Indigenous Australia.

**International Registered Report Identifier (IRRID):**

DERR1-10.2196/68892

## Introduction

Comprehensive primary health care (CPHC) is the delivery of essential health care services from the first point of contact, enabling equitable access to health and well-being for all residents in a community [1]. In the context of Australian Aboriginal Community Controlled Health Services (ACCHSs), CPHC has four domains for delivering core services: governance, clinical services, community health promotion and empowerment, and policy direction and partnerships [2]. In common with primary health care services, such as those delivered by Northern Territory (NT) Health, ACCHSs deliver a broad range of clinical services. However, ACCHSs have governance structures that support the expression of self-determination and offer services that include community development, advocacy to improve determinants of health that are outside the health system, and fostering partnerships to optimize action addressing those determinants. CPHC is characterized by the achievement of more equitable health outcomes in line with the Alma-Ata Declaration of 1978 on primary health care [3,4]. CPHC is also the most inclusive and cost-effective approach to delivering health care services and involves identifying local health needs and responding to health provision gaps [1]. Strong CPHC systems are therefore essential for optimizing health outcomes for high-needs, dispersed, and marginalized communities.

Remote Aboriginal and Torres Strait Islander (respectfully referred to as Indigenous throughout this protocol) Australians experience the heaviest burden of ill health and the highest rates of preventable hospital admissions and potentially avoidable deaths of all Australians [5]. Overall, Indigenous Australians have 2.3 times the burden of disease compared to the non-Indigenous population [5]. Responding adequately to this burden of disease and the associated need for CPHC in remote Indigenous communities of the NT is challenging [6]. The NT spans a large geographical area (1,347,791 km^2^), all classified as rural or remote, and population density is sparse [7]. In Australia, geographical remoteness has been associated with long-standing health workforce issues, including poor supply, low retention, and high turnover, with associated adverse impacts on access to CPHC, especially continuity of care [8-11]. In addition, high proportions of nurses are employed on casual or short-term agency contracts [12]. On average, only 20% of remote area nurses and Aboriginal health practitioners (AHPs) remain working in the same clinic 12 months after commencing their employment [8,12,13]. While the use of temporary staff provides a short-term solution to address dire need, there are trade-offs regarding the skills and suitability of these staff, with consumers preferring to have their complex chronic conditions managed by health care professionals they know [8,9,12]. High mobility of remote populations is also a feature of remote communities and is a further barrier to continuity and access to care.

Digital health technologies (DHTs) such as telehealth, wearable devices, and mobile health apps can help to address barriers to CPHC for people living in remote Indigenous communities as well as support remote area health care professionals [6,14]. Telehealth has been shown to reduce the need to travel, decrease waiting periods to see a clinician, and improve continuity of care [15,16]. DHTs can also increase flexibility of employment and provide enhanced training opportunities for remote clinicians [17], thereby promoting workforce retention. DHTs may also improve communication between different service providers [17], which can improve coordination of health care, improve decision-making, and strengthen communications between specialists and primary health care providers. DHTs also have the potential to increase cultural security and minimize disparities in access to health care [18]. For instance, a recent systematic review reported that in some circumstances, Indigenous people may feel more comfortable using telehealth and thereby avoid embarrassment, which might arise from a face-to-face interaction with a health provider who lives in their community [19]. Nevertheless, Indigenous peoples are diverse and have different needs. For cultural safety to be in place, trusting and respectful relationships must first be created. Therefore, the vision of the NT Virtual Care Strategy [6] is that DHTs will facilitate improved access, result in greater choice for consumers, reduce travel time to get to appointments, and increase convenience for consumers.

Several studies have developed and applied digital tools such as apps and programs to promote health and wellness in rural and remote communities, but these are often not developed in collaboration with communities and, when they have been, are rarely evaluated [20,21]. In the NT, DHTs being used include telehealth (audio with video), electronic patient management information systems, and a range of mobile apps. However, despite existing use and the potential for more widespread adoption of DHTs, it is not well understood which DHTs might work well, how, and for whom to improve access to CPHC for remote Indigenous populations in the NT. While some health services in the NT have used DHTs to deliver CPHC and support follow-up of specialist care, DHTs generally remain underutilized and have neither been systematically implemented nor evaluated [20,21]. The use and type of DHTs in the NT have primarily been driven by providers’ preferences [14], with limited focus on consumer preferences and experiences. Likewise, there is little evidence of consumer involvement in the design and delivery of health care using DHTs in remote Indigenous settings elsewhere in Australia, which stand to benefit most from this technology. In light of these important gaps, this research will co-design, implement, and evaluate the use of DHTs to strengthen the evidence about how DHTs can best support access to CPHC for remote Indigenous communities, considering consumer and remote health staff preferences. This protocol is reported in line with the iCHECK-DH (Guidelines and Checklist for the Reporting on Digital Health Implementations) reporting guideline, with some adaptations in subheadings [22].

## Methods

### Setting

This project is being conducted (July 2022-June 2025) in two remote Indigenous communities in the NT, where health care is provided by either NT Government-run health services or ACCHSs. Health services across the NT were invited to express their interest in participating in the project. A project advisory committee, consisting of key decision-makers from the NT and Commonwealth Health Departments, ACCHSs, and stakeholder partners, selected the sites based on community size, remoteness, location (Top End, Central Australia), health service delivery and governance model (government, ACCHS), and estimated readiness to engage with DHTs. This approach sought to maximize the opportunity to evaluate and compare the findings from diverse communities with different governance structures. The clinics in these communities are mostly staffed by remote area nurses, AHPs, and nonclinical administrative staff based in the community, with visiting (and sometimes telehealth) services provided by general practitioners, Allied Health Professionals, and various medical specialists. The NT has unique cultural, social, and language considerations, with 26.3% of the population identifying as Aboriginal or Torres Strait Islander (compared to 3.2% across Australia) [23]. In remote and very remote communities of the NT, more than 90% of the population identify as Aboriginal, and for many, English is not their first language [23]. The participating communities in our project meet this unique NT profile: they are small (<400 population), very remote, and have a predominantly Indigenous population. They face numerous health care delivery challenges and have differing usable technological infrastructure, such as the presence of a 4G mobile network, the presence of microwave, satellite, or cable internet, and the presence of community Wi-Fi.

### Theoretical Frameworks

A participatory action research (PAR) methodology actively involves key stakeholders, including patients (consumers), health care providers, caregivers, and community members in the research process. This methodology emphasizes collaboration, shared decision-making, and engagement of those directly affected by the research [24,25]. The intention is that a network of participants who are actively engaged in the project can help bridge the gap between research and practice to create sustained system change.

**Figure 1:**
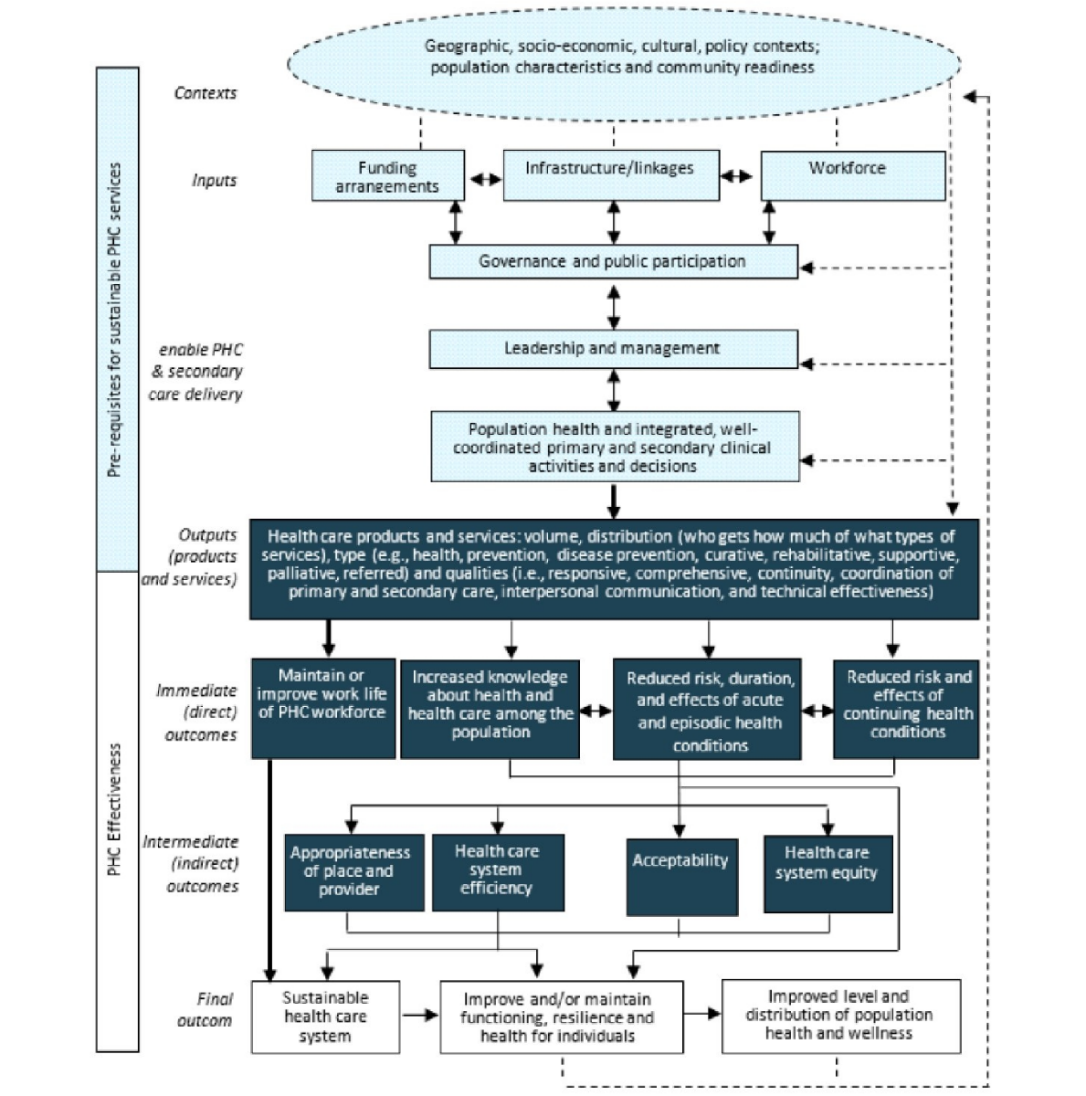
An evidence-based logic model for sustainable comprehensive primary health care for small, rural, and remote communities. NT: Northern Territory; PHC: primary health care.

We will apply three theoretical frameworks in the design, implementation, and evaluation of DHT solutions in the 2 communities. First, an overarching logic model (Figure 1 [26]) for CPHC will be used to provide a systematic approach for data collection across the different components of a CPHC system (context, inputs, enablers, outputs, and immediate, intermediate, and outcomes), and the contribution of each component will be monitored against relevant outcome measures [27].

Second, the six dimensions of the modified theory of access—acceptability, accommodation, affordability, accessibility, availability, and awareness (including digital health literacy)—will be used to evaluate the impact of DHTs in providing enhanced access to CPHC [28,29].

Third, a realist approach will be used to explore which DHTs work, for whom, and under what circumstances [30,31]. Realist evaluations go beyond simply determining whether an intervention is effective or not; they provide insight into underlying mechanisms and the context of causal relationships between interventions and outcomes.

This tri-part approach is likely to be especially useful to unpack the complex interplay of factors operating within remote and Indigenous health care environments [30,32].

Specific research questions include:

1. What technologies are available to support CPHC, including prevention and health promotion programs, in remote communities?
2. How can existing technologies be modified or enhanced to support CPHC, and what new technologies are needed?
3. What are the consumer and provider preferences for the use of digital technologies to support CPHC in remote communities?
4. How can technologies be used to promote access to care and support a holistic approach to social and emotional well-being in remote communities?
5. How can digital technologies support coordination of, and access to, CPHC in a highly mobile population, as well as collaboration between providers?
6. What skills are required for remote providers to use digital technologies to support CPHC in remote communities?
7. What is the impact of deploying different digital technologies to support CPHC on consumer and staff experiences in remote communities?
8. What are the potential cost consequences of deploying different digital technologies to support CPHC in remote communities?
9. How can the deployment of digital technologies improve the efficiency of CPHC in remote communities?

### Project Co-Design, Implementation, and Evaluation

The project will be conducted in multiple phases, including investigation and co-design of DHTs with consumers and health service staff, iterative implementation of different DHTs in two community sites, and evaluation of these DHTs on consumer and staff experiences and access to CPHC (refer to Figure 1).

#### Preimplementation Activities and Co-Design of Digital Health Interventions

A desktop review will identify existing DHTs being applied in the NT and elsewhere that can be applied to support CPHC in remote Indigenous communities. A concurrent realist review will develop initial program theories about how and for whom DHTs affect access to CPHC in remote Indigenous communities [30,33]. Initial program theories developed from the existing literature will be refined using data collected from thought leader interviews (refer to the interview guide in Multimedia Appendix 1). Some of these theories may subsequently be tested during the evaluation phase (depending on preferences for DHTs to be implemented).

A technology-enabled patient journey map will be prepared and refined iteratively for use during interviews and community interactions. It will be a tool to assist with identifying the facilitators and barriers for remote residents accessing CPHC during different stages of their health care journey.

Initial community-engagement activities will entail consulting local elders in the two selected sites. This will inform the employment of local community-based Indigenous researchers. Elders will be consulted throughout the project. Male and female local community-based Indigenous researchers will be employed at each site. These researchers will be key to ensuring locally responsive and culturally appropriate data collection and for developing and maintaining relationships between the research team and local stakeholders [34]. Aboriginal researchers will actively participate in all stages of the project and all phases of the data lifecycle, including data tool creation, data collection, access analysis, co-design, analysis, interpretation of results, and dissemination of findings, including publications and presentations to key stakeholder groups.

Baseline data will be collected on general communities’ views on the use of technology for health and wellness, digital health coverage [35], eHealth literacy [36], and consumer satisfaction with current use of telehealth to access health services and group health promotion sessions [37-39].

Qualitative semistructured interviews and focus group discussions (FGDs) with consumers and providers will be undertaken to explore preferences for DHTs and to inform the attributes of DHT interventions [40,41]. Community-based researchers will actively identify and recruit participants for FGDs and interviews. We will use a yarning method to conduct these FGDs and interviews. Discussions will be led by community-based Indigenous researchers to ensure that FGDs and interviews are conducted in a culturally safe way. The information obtained from the FGDs and interviews will be used to identify the key attributes of DHT interventions, which will inform the preimplementation quantitative survey. The survey will quantify the best and worst attributes of DHTs using best-worst scaling (BWS), digital technology coverage, eHealth literacy, and satisfaction of consumers who have used DHTs. The BWS will quantify consumer and local primary health care staff members’ priorities for key attributes of DHTs and any differences in preferences among different groups of respondents. These findings will be used to further refine and target DHT development and implementation [41]. Local elders will be consulted at each stage of the project, ideally before employing local community-based Indigenous researchers and before each stage of the project.

#### Implementation

Information regarding the candidate DHTs identified during the co-design phase will be presented to the consumers, local health staff, and the leaders in government and community sectors. An integrated set of candidate DHTs will be implemented based on their feasibility and anticipated impact on access to CPHC. Interventions will be tailored to the local context using the PAR design approach led by community-based researchers who will support intervention implementation and lead the collection of feedback.

#### Evaluation

Postintervention data will be collected as the interventions are implemented and iteratively refined. We will use quantitative administrative data and qualitative data from interviews and FGDs with consumers and stakeholders about their experiences with DHTs in order to identify what works for whom, in what context, and by which mechanism [30].

An economic analysis will investigate the incremental costs of implementing DHTs and the associated benefits. The incremental resource use will be calculated based on different patterns of digital solution uptake in terms of additional resources required, and staff costs are based on assumptions around how many extra staff hours would be required per patient per unit of time. Whether a cost-effectiveness or cost-benefit analysis is conducted will be determined through consultations with key stakeholders.

#### Scalability Analysis

Scalability analyses will be conducted in two additional communities, using a previously published framework [42]. The objectives of such analysis will be to (1) identify core and adaptable features of implemented DHTs and (2) explore the feasibility of scaling DHTs to additional community sites, including cost implications. The scalability analysis will enable the evaluation team and participating organizations and their respective health boards to assess the suitability of the developed DHTs for scale and further implementation. This component will have strong translational implications.

### Participants

Community partnership is an important aspect of this project. It is crucial to understand how remote communities experience access to CPHC and how DHTs may improve or deter their access to and experiences with health services. A total of 6 stakeholder groups were identified as crucial partners in this project (refer to Table 1 for details). Briefly, group 1 includes community leaders, group 2 includes consumers, and group 3 includes health service staff, health board members, and clinical and nonclinical staff at the participating sites. Likewise, group 4 includes project partner organization staff, group 5 includes technology provider organization staff, and group 6 includes other stakeholders and thought leaders. Participants must be aged 18 years or older and able to provide informed consent.

This is a multi-institutional collaboration that includes government and industry partners [43]. The key partners include the Digital Health Cooperative Research Centre, the Commonwealth Department of Health and Aged Care, the NT Government, the NT Primary Health Network (NTPHN), the Aboriginal Medical Services Alliances NT, Healthdirect, the Australian Digital Health Agency, the Menzies School of Health Research, and the University of Sydney. Representatives from each partner provide substantial research and industry input at all stages of the project.

**Table 1:** Research participants, recruitment, and data collection methods for each phase of the project, evaluating the design and implementation of digital health technologies in two remote Indigenous communities of the Northern Territory, Australia.

Data source		Example participants	Sample	Recruitment method	Data collection method	Time
**Primary data**						
	Workshops with remote NT^a^ health service providers	GPs^b^, allied health professionals, remote area nurses, and health service managers	60	Invitation; convenience sampling	Group discussions	Preimplementation
	Group 1: community leaders at the two participating sites	Traditional owners, mayors, religious and cultural leaders, and local council board members	8	Purposive sampling via local contacts	Individual interviews	Co-design, implementation, and evaluation
	Group 2: consumers at the two participating sites	Consumers (or their carers) who have used local health care services	100	Purposive sampling to ensure representativeness	Survey administered in person (digital technology coverage, best-worst scaling survey, consumer satisfaction, and eHealth literacy)	Co-design, implementation, and evaluation
	Group 2: consumers at the two participating sites	Consumers (or their carers) who have used local health care services	40	Purposive sampling to ensure various perspectives are included	Individual interviews and focus group discussions	Co-design, implementation, and evaluation
	Group 3: health service staff at the two participating sites	CEOs^c^, managers, board members (where applicable), clinical staff (eg, doctors, remote area nurses, and Aboriginal health workers), and nonclinical staff (eg, drivers and administrative staff)	25	Purposive sampling from the local sites	Individual interviews and small group discussions	Co-design, implementation, and evaluation
	Group 4: partner organization staff	NT health staff members and other partner organizations such as AMSANT^d^ and NT PHN^e^	25	Purposive and snowball sampling	Workshop, small group discussion, individual interviews, and system usability surveys	Preimplementation
	Group 5: technology provider organization staff (nonpartners)	Current staff of technology providers such as NBN Co^f^ and Telstra	5	Purposive and snowball sampling	Meeting minutes and documents, interviews	Implementation
	Group 6: other stakeholders and thought leaders	Staff at other remote clinics or those who have an in-depth understanding of the remote CPHC^g^ context	25	Purposive and snowball sampling	Individual interviews and workshops	Preimplementation and co-design
**Secondary data**						
	Comprehensive primary health service usage data	All consumers of participating health care services	All consumers	NT Health^h^ and ACCHSs^i^	Clinic records and activity, CPHC service utilization data, hospitalization data, and patient travel data	Baseline and end-line (01/01/2022 to 02/28/2025)
	Economic data	The key data source will be health personnel and technology-related costs.	All expenditure relevant to DHTs^j^	NT Health and ACCHSs	Expenditure and personnel data from participating institutions. Obtain data from NT Health for the participating clinic and from the ACCHSs	Baseline and end-line (01/01/2022 to 02/28/2025)

^a^NT: Northern Territory.

^b^GP: general practitioner.

^c^CEO: chief executive officer.

^d^AMSANT: Aboriginal Medical Services Alliances Northern Territory.

^e^NT PHN: Northern Territory Primary Health Network.

^f^NBN Co: National Broadband Network Company.

^g^CPHC: comprehensive primary health care.

^h^NT Health: Northern Territory Department of Health.

^i^ACCHS: Aboriginal Community Controlled Health Services.

^j^DHT: digital health technology.

### Data Collection

Table 1 provides a summary of data sources used at each stage as the project progresses.

### Primary Data

Initial workshops and key informant interviews with thought leaders will be used to develop primary health care journey maps that depict how different DHTs are being used. Barriers and enablers of access to CPHC and the role of DHTs in improving access will be discussed to identify initial outcomes and mechanisms.

Interviews with stakeholders will be used to explore their perspectives on various barriers and enablers of access to CPHC and the role of DHTs in improving various aspects of access. Focus group discussions with consumers in the communities will explore key challenges and identify their preferences. A baseline survey, including the BWS survey, will collect data on DHT coverage, consumer experiences with DHT use [37,38], eHealth literacy [36], and consumers and health staff preferences for DHT implementation [40,41].

Once preferences of consumers and health care providers regarding DHTs are identified and feasible DHTs are implemented and optimized, focus group discussions, interviews, and surveys will be used to collect data about consumer use and satisfaction. This will be an iterative process and will inform scalability to other remote Indigenous communities.

### Secondary Data

Secondary administrative data will be retrieved from multiple sources, including CPHC data at the two intervention sites, such as utilization data, hospitalization, and patient transport data. These analyses will complement scalability and economic analyses. Expenditure and personnel data (eg, workforce composition and supply) required for economic analyses will be obtained from the participating organizations. Further secondary data needs will be determined once the DHTs are prioritized (refer to Table 1).

### Data Analysis

#### Qualitative Data

All interviews, focus group discussions, and workshops will be audio-recorded (if acceptable at the time) and transcribed; detailed field notes will also be used to inform analysis and understanding. Transcripts will be thematically analyzed [44,45] using a combined inductive and deductive approach and the six phases suggested by Braun and Clarke [45], that is, familiarizing with the data, initial coding and agreeing on codes, identifying themes, reviewing them, finalizing themes, and writing the results. The analysis will also be informed by realist approaches [30] to examine what works for whom and in which contexts. Data collection and analysis will be guided by multiple theoretical frameworks. For instance, the modified theory of access [28,29] will be used to identify the barriers faced by remote communities in accessing CPHC. The Systems Engineering Initiative for Patient Safety (SEIPS) framework will be used to identify system factors that influence how care is delivered in digitally enabled models of care and lead to better outcomes for patients and remote health service staff [46]. Due to the co-design nature and unique complexities of each digital health initiative, some flexibility remains in adapting theoretical frameworks specific to each initiative. Lumivero NVivo software will be used during data analysis to identify barriers and facilitators to DHT across different domains of access and the mechanisms by which interventions impact access to CPHC for different groups of consumers. Case studies will be used to understand what DHTs work (or do not) for CPHC in remote Indigenous communities, for whom, in what circumstances, and by what mechanism to improve access to health care [30,31].

A total of two community-based Indigenous researchers from each site will be involved throughout the project. They will accompany other experienced qualitative researchers to conduct each stage of data collection and analysis. Various data sources, such as consumer, provider, observation, and quantitative data, will be used to triangulate the findings. Multiple researchers will be involved in coding, refining, and summarizing data. Results will be shared with community members and the respective community-based researchers to validate the findings. The community engagement and data collection will happen over many months; therefore, trust building and longitudinal relationships are kept at the center of the data collection process.

The researchers come from diverse backgrounds, with expertise covering health services, clinical practice, remote care, digital health, human factors, and community engagement. The project leaders are non-Indigenous; however, they have spent significant time in the Indigenous health space. The community-based researchers will be the key bridge between the community and the rest of the project team. This will allow the non-Indigenous researchers to remain conscious of their viewpoints and biases and remain intentional to ensure the voice of participants is heard while interacting with the community, collecting and analyzing data, and presenting the findings. Findings of the study will be shared with key community stakeholders and community-based researchers to confirm that it aligns with the community’s view.

#### Quantitative Data

Pre- and post-intervention data will be compared to investigate the changes in the use of primary health care services. Descriptive statistics using frequency tables and visualization, along with, where possible, univariate and multivariable regression analyses, will determine the association between the DHT interventions and revealed access to CPHC.

The economic analysis will present disaggregated incremental costs and consequences of different DHT uptakes relative to the status quo. In addition, changes in patient satisfaction and outcomes, use of primary care, hospitalizations, and patient transport will be assessed to determine the impact on incremental costs and outcomes of changing values of cost or outcome variables and alternative ways of delivering care.

#### Data Management

A central data repository of quantitative and qualitative data will be maintained on a secure university server. These data will also be stored on the university and health service organizations’ password-protected computers of individual researchers who have ethical clearance to access these data during the study. Collaborators will ensure that their IT network infrastructure complies with Australian IT security standards and the General Data Protection Regulation.

Principles of Indigenous data sovereignty will be adhered to, including the involvement of Aboriginal people in all stages of data acquisition, use, and reuse.

### Project Governance

This project is situated within the governance of the Strengthening Our Health System Strategy, an established collaboration of Aboriginal Medical Services Alliances NT Health (the state health department), and NT Primary Health Network. It focuses on crucial aspects of the NT Virtual Care Strategy [6] relating to improving equity, delivering culturally safe services, and providing support, resources, and guidance for staff about how and when digital health care is effectively provided. It is overseen by a Project Control Group and a Project Advisory Group, each having representation across all partner organizations and the Commonwealth Department of Health. Around 3 additional working groups (the research working group, project operational team, and technical working group) hold responsibilities for guiding research activities, ensuring smooth project delivery, and navigating technology issues in the implementation and adaptation of DHTs for CPHC in remote NT.

This implementation project has Indigenous leadership and representation, including as chief investigators, and at all stages, including design and implementation. This project also uses 1 male and 1 female community-based Indigenous researcher at each study site. The community-based researchers are mentored by experienced research team members and have been provided with training on ethical conduct in research, data collection, and analysis, and they provide reciprocal cultural mentoring for non-Indigenous research team members.

### Knowledge Translation

Knowledge translation is an integrated feature of the project and is of particular interest to stakeholders and other partners in the project. The governance structure enables rapid feedback on project developments and outcomes. Sharing findings with stakeholders, health services, funders, and executive groups (eg, NT Health and the Commonwealth Department of Health) will be prioritized to inform the scalability and suitability of DHTs for CPHC in other remote Indigenous communities. Customized reports will be provided to each of the stakeholder groups and participating services. A one-page, plain-language report will also be distributed to the participating communities and all NT remote health services. The lead researchers and local community-based Indigenous researchers will present findings to key members of the community. Where necessary, Aboriginal community-based researchers will help interpret the findings in the local language. Dissemination will include peer-reviewed publications and presentations at conferences and professional workshops.

### Ethical Considerations

The project was reviewed and approved by the Human Research Ethics Committee of the Northern Territory Department of Health and the Menzies School of Health Research (HREC approval no 2022-4275). Administrative approval was also obtained from the Research Governance Office, NT Health (EFILE2023/8720), and from the Boards of any participating ACCHS. Research project approval was also sought from the respective land councils, and appropriate travel and work permits were obtained for each site to support community-based research activities.

## Results

As of November 2024, more than 70 consumers, 17 thought leaders, and 20 health care professionals have been interviewed to assess the feasibility of various digital health initiatives to improve access to health care in the two implementation sites. Researchers have spent more than 50 days at each implementation site. Several digital health initiatives have been co-designed and optimized, which include telehealth from the primary health care clinics to outpatient services, telehealth for aged care allied health services, an SMS text messaging program on selected topics identified by the community, and exploring the feasibility of teledentistry. An economic analysis is also underway to help inform the financial benefit (or loss) of the initiatives. Initial findings from these sites have informed scalability assessments in two additional sites. Data analysis is progressing. The findings will be reported in reports and peer-reviewed publications.

## Discussion

### Principal Findings

This project aims to optimize the use of DHTs to improve access to CPHC in remote Indigenous communities. DHTs, including telehealth, have been used ad hoc or as pilot projects rather than for mainstream ongoing health care delivery [47]. While there are promising findings from other studies in the NT [14,16], to the best of our knowledge, our project will be the first to co-design DHT interventions with consumers and remote health providers and to systematically evaluate the acceptability and effectiveness in remote Indigenous communities.

Key findings from this project will include community preferences and priorities for digital health, the co-design process of the digital health initiatives, and learnings from implementation and evaluation. The findings will provide evidence on the impact of digital health initiatives on access to health care and remote community and health provider experiences. Barriers and facilitators to implementing these initiatives will help inform learnings for future DHT implementations in remote communities.

The involvement of multiple stakeholders, including consumers, health service providers, technology partners, and policy and funding partners, is critical to improving the knowledge about and skills for using DHTs to provide CPHC in remote communities [24,48]. The use of one DHT (telehealth) to improve access to CPHC has been examined in remote NT, and it has been reported that the doctors were able to provide more clinical care because less of their time was consumed traveling to each community [14,16]. This project considers a broader range of DHTs, extending beyond telehealth to include, for example, SMS text messaging for appointment reminders, medication reminders, health promotion messages, community health portals, and use of the Australian Government’s My Health Record. The intention is to improve access to CPHC, promote equity, and improve health outcomes in the two remote communities through improved continuity of care and fewer avoidable hospitalizations and patient transfers. Effective and acceptable CPHC also capitalizes and contributes to increased trust and long-term relationships with health service providers to increased cultural security, greater convenience in receiving health care, a reduction in health care disparities, and stronger community engagement in health management and well-being [6,14].

The economic analysis is unique, as previous studies have not reported such systematic evaluations. The virtual care strategy of NT Health [6] has reported that digital health has the potential to save AU $6.3 million (US $4.095 million) on annual travel costs and AU $950,000 (US $617,500) on the cost of appointment cancellations in the NT (1 AU$ =0.65 USD). However, these estimates are not based on community-based studies from the NT. Our economic evaluation intends to show the financial benefit at the local level for the clinic and patient, which will inform decision-making about implementing DHTs in CPHC.

Unintended consequences are not uncommon in the digital health space, and one such risk is the additional administrative and clinical workload that the resident staff may bear and the concern for burnout [17,47]. The co-design and PAR approach is expected to help identify such issues proactively and address them on time. For instance, additional workload needs to be accounted for when telehealth consultations with outpatient hospital services or health promotion sessions are offered in the community [49].

A second issue is managing the “digital divide” [50,51]. Cautious and informed DHT identification and implementation will minimize the impact of broad-stroke DHT interventions that widen the gap in access to CPHC experienced by disadvantaged populations who may not have digital devices, connectivity, or digital literacy or skills for such consultations [52,53]. The realist approach, by identifying how DHTs work and who needs support, why, and in what circumstances within the available provisions, adds further critically important information regarding approaches to DHT implementation in marginalized populations such as those living in remote Indigenous communities [30,31].

A third issue is managing perceptions that telehealth may be “a cheaper option” for CPHC in remote communities [10]. In an ideal situation, the same clinical staff would always be available face-to-face for consultations, but in many remote communities, this is not realistic due to high staff turnover, declining general practitioner training enrollments, vast geographical distribution of the smaller communities, and the inequitable distribution of resources [8,27,54]. This project will identify opportunities to implement DHTs that do not take away existing face-to-face services but rather complement and enhance CPHC in remote communities. The DHT interventions may be catalysts to improve health and well-being in remote Indigenous communities.

### Limitations

There are important limitations to consider in this study. Due to the small remote community population sizes, the sample included in the studies will have limited statistical power but will have inclusive consideration for the remote communities. Poor digital connectivity and high staff turnover, including changes in the health service administration, are also beyond the control of our project team. The use of secondary administrative data that have not been collected for the express purpose of the study is likely to present additional challenges. For example, the ACCHSs and government health services use different patient information management systems, limiting the ability to link datasets and the comparability of data available at different sites. Despite its strengths, the PAR approach is time-consuming, needs additional efforts to focus on processes, and has the potential to lead to friction among the stakeholders due to competing priorities [24,25]. While it can be counterproductive if not handled with care and expertise, this has been accounted for in the project design and development strategies, which are founded on the considerable expertise of the research team. The credibility and local ownership offered by the PAR approach outweigh these limitations.

### Conclusions

Digital innovations have affected every aspect of our lives. It is unlikely that the current health system and health service delivery can remain isolated from such a rapid proliferation of innovative DHTs. This project provides a unique opportunity to optimize and systematically evaluate key digital health interventions. It will also highlight why DHTs work for some but not for others, identifying risk groups to create appropriate supports within the community. Furthermore, the economic analysis will help the local health board and other stakeholders make an informed decision to adopt, refine, or reject DHTs in the future. In conclusion, the project has the potential to substantially improve access to CPHC services for the remote Indigenous communities of the NT. Such improved access will help improve the health outcomes of those living in remote communities.

## Appendix

Multimedia Appendix 1 Interview guide sample.

## Abbreviations

ACCHS: Aboriginal Community Controlled Health Service
AHP: Aboriginal health practitioner
BWS: best-worst scaling
CPHC: comprehensive primary health care
DHT: digital health technology
iCHECK-DH: Guidelines and Checklist for the Reporting on Digital Health Implementations
NT: Northern Territory
PAR: participatory action research
SEIPS: Systems Engineering Initiative for Patient Safety
