# Supplementary material for: Optimizing Digital Solutions to Improve Access to Comprehensive Primary Health Care Services in Remote Indigenous Communities: Protocol for a Participatory Action Research Project
Source: JMIR Res Protoc. 2025 Sep 17;14:e68892. doi: 10.2196/68892 (PMC12489406; doi:10.2196/68892)
Supplement: Multimedia Appendix 1 [file resprot_v14i1e68892_app1.docx]

**Interview Guide**

**Thought Leader Interview**

This is a guide only. Interviews will be semi-structured covering these topics and related issues as they arise.

- Introduce interviewer
- Introduce study
- Review information statement
- Discuss confidentiality, audio-recording and use of information
- Sign consent form

1. Can you tell me about yourself and the work you do in Northern Territory?
2. Are you able to tell me about the challenges you/health professionals might face to ensure your patients can access primary health care in remote NT? Where on the journey do these challenges occur?
3. I’d like to look at the other side… what works well for ensuring access to primary health care in remote NT? Where on the journey are things working well?

- Why do you think that’s not working? (ie. identify mechanisms, contexts)
- How do you know it’s not working – what’s happening? (ie. identify unwanted outcomes of importance)

*(*Interviewer to prompt about: accessibility, affordability, availability, acceptability, accommodation and awareness).*

- How do you navigate/mitigate/work around this challenge? What helps it to work?

1. I’d like to look at the other side… what works well for ensuring access to primary health care in remote NT? Where on the journey are things working well?

- Why do you think it works well? (ie. identify mechanisms, contexts)
- How do you know it’s working well? (ie. identify outcomes of importance)

1. We’ve talked about your challenges as a health provider, but what might be some of the challenges remote patients might face in accessing primary health care in remote NT? Where in the journey do these challenges for patients occur?

- Why do you think that’s not working? (ie. identify mechanisms, contexts)
- How do you know it’s not working – what’s happening? (ie. identify unwanted outcomes of importance)
- What might help it work?

1. I’d like to look at the other side… what works well for patients to ensure their access to primary health care in remote NT? Where on the journey are things working well?

- Why do you think it works well? (ie. identify mechanisms, contexts)
- How do you know it’s working well? (ie. identify outcomes of importance)

1. We’re particularly interested in the space for digital technology to help improve delivery and access to comprehensive primary health care…

What digital technologies are you or other health professionals already using in remote NT to provide primary health care? (eg. Video consult, tele consult, e-scripts, electronic medical records, remote monitoring devices, smart phone apps. etc.)

- At what stage in the patient journey are these technologies used?
- How well (or poorly) does each digital tech work? (outcomes)
  - What might help the digital tech to work?
- Why is that? What was good/not so good about it? (mechanisms, contexts)
- What’s the experience like for you and your colleagues?
- What do you think the experience is like for patients?

1. What can be done using digital technologies that might help address the challenges we’ve been talking about?

- Which of these might have the biggest impact? Why?
- Which is the most feasible? Why?

1. Thinking more broadly about using digital technologies to improve access to PHC;

- What were/are the key challenges in using digital technology to ensure access to primary health care for patients? *(if they are not using digital tech, explore on anticipated challenges)*.
- How do you think these challenges could be resolved? (new mechanism/intervention)
- What would be the impact?

1. In an ideal world, what would access to primary health care using virtual care/digital health look like in remote NT in five years?

- How would it work ? (eg. mechanism)
- What do you think could feasibly help us move towards that vision?
